# A randomized controlled trial of the effects of whole grains versus refined grains diets on the microbiome in pregnancy

**DOI:** 10.1038/s41598-022-11571-4

**Published:** 2022-05-07

**Authors:** Haipeng Sun, Pamella Yamada, Alexandra Paetow, Michael Chan, Alan Arslan, Rikard Landberg, Maria Gloria Dominguez-Bello, Bruce K. Young

**Affiliations:** 1 Department of Biochemistry and Microbiology, Rutgers University, New Brunswick, USA; 2 Department of Obstetrics and Gynecology, New York University Langone Health, 462 First Avenue, Building D, Room 572, New York, NY 10016 USA; 3 Division of Food and Nutrition Science (RL), Department of Biological Engineering, Chalmers University of Technology, Gothenburg, Sweden; 4 Department of Anthropology, Rutgers University, New Brunswick, USA; 5 Institute of Food, Nutrition, and Health, Rutgers University, New Brunswick, USA

**Keywords:** Microbial communities, Nutrition

## Abstract

Dietary whole grain consumption has been postulated to have metabolic benefits. The purpose of this study was to compare a pregnancy diet containing 75% of total carbohydrates as refined grains with a diet of 75% of total carbohydrates as whole grains for pregnancy outcomes and effects on the microbiome. Gestational weight gain, glucose tolerance and newborn outcomes were measured on 248 enrolled compliant women from whom a subset of 103 women consented to give 108 vaginal and 109 anal swabs. The data presented here are limited to the patients from whom the vaginal and anal swabs were obtained in order to study the microbiome. A microbiome—*16SrRNA* survey—was characterized in these samples. Samples and measurements were obtained at the first obstetrical visit, before beginning a prescribed diet (T1—baseline) and after 17–32 weeks on the prescribed diet (T3). Food frequency questionnaires and total plasma alkylresorcinols were used as a measure of whole grain consumption. There were no dietary differences in maternal weight gain, birth weight, or glucose tolerance test. Mothers consuming the whole grains diet showed a trend of gestational decrease in vaginal bacterial alpha diversity, with increasing Lactobacillus-dominance. No significant difference was observed for the anal microbiome. The results suggest that diet modulations of the vaginal microbiome during gestation may have important implications for maternal and neonatal health and in the intergenerational transfer of maternal microbiome.

**Trial registration: **ClinicalTrials.gov Identifier: NCT03232762.

## Introduction

Recently the whole grain content of the carbohydrates consumed in pregnancy has been considered to influence outcomes such as maternal weight gain, large for date infants and childhood obesity^1–4^. Current dietary guidelines for healthy eating recommend at least 50% whole grains in nonpregnant women, but the evidence for recommending that in pregnancy is not robust^5^. The importance of pregnancy diet is emphasized by the Barker hypothesis, which states that there is a profound effect of intrauterine nutrition on outcomes from neonates to adulthood^6–8^. Although many guidelines for diet in pregnancy have been promulgated, the evidence for them derives from epidemiologic data with few interventional trials, and none have evaluated effects on the anal and vaginal microbiome. In fact, the U.S. Consensus Report on Healthy Eating for the nonpregnant population finds inadequate evidence for a direct contribution of added sugars (included in the refined grains food category) to obesity or heart disease^9–12^. We report a subset of the patients who had microbiome data from a large randomized trial^13^ comparing the effects of refined and whole grains diets in normal pregnant women.

Grain foods contribute to nutrient density and are meaningful sources of iron and vitamins in children^14^. However, there are differences between whole grains and refined grains in their contributions of specific nutrients and dietary fiber^14^. Nutritional references to refined grains and whole grains are standards defined in the National Health and Nutrition Examination Survey (NHANES) studies^14^. Most reports are statistical analyses based on food frequency questionnaires (FFQ). These data are observational and not based on clinical trials. There are few interventional studies, and none that is specific for grains consumption^15–21^. Consequently, there is a need for dietary interventional studies to provide more robust data regarding pregnancy. There are no known risks to such interventions, and the potential benefits are a reduction in pregnancy complications and in childhood obesity^17^. Changes in the maternal microbiome in pregnancy are known, as are the effects of cesarean birth on the neonatal microbiome^22,23^. These have significant relationships to the immune system and metabolism, amplifying the Barker hypothesis cited above. However, the effect of dietary differences for normal pregnant patients’ microbiome has not been reported.

We sought to evaluate the effects of a diet with different proportions of total carbohydrates, as either 75% refined grains or 75% whole grains, on pregnancy associated changes in the maternal microbiome and pregnancy outcomes.

## Results

This randomized interventional study enrolled 303 pregnant women (from August 2017 to April 2019) in the first and second gestational trimester. From a subpopulation of 103 mothers, we collected 109 anal and 108 vaginal swabs and compared the data from this subset. The swabs were obtained in the first and second gestational trimester (T1 baseline before beginning a prescribed diet, gestational ages 8–23 weeks), and third trimester (T3 in the third trimester, after 17 to 32 weeks of dietary intervention; Supplementary Table 1, Supplementary Fig. 1). In total 5,113,342 high quality 16S V4 region reads were generated, and the average number of reads per sample was 27,054, after applying a rarefication to 8000 reads, 3 samples (1.6%) which contain less than 8000 reads were excluded.

### The vaginal microbiome

**Figure 1:**
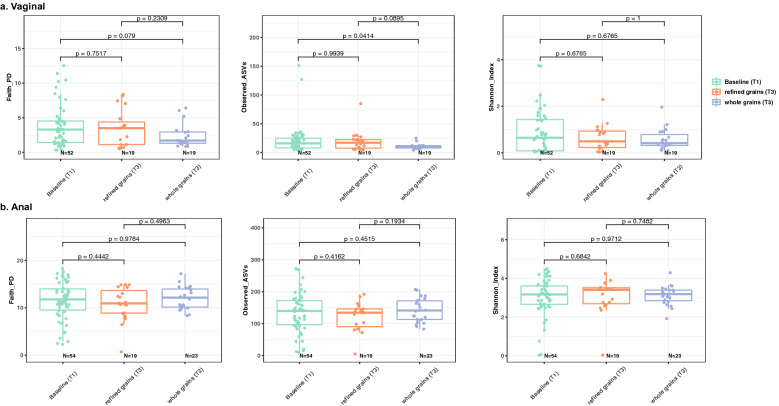
Alpha diversity in the vaginal and anal microbiota of 103 mothers. Faith PD, Observed ASVs, and Shannon index was presented respectively, Kruskal–Wallis test performed between the three groups. (**a**) Vaginal microbiome. (**b**) Anal microbiome.

*Lactobacillus* dominated the vaginal microbiome during gestation, contributing to about 80% of the mean relative abundance (Supplementary Fig. 2a) in both diet groups. Comparison to the baseline combining all the samples at T1 was made for each group at T3. The structure of the microbiome was relatively stable and showed a statistically significant difference with lower alpha diversity in the whole grains samples at T3 (Observed ASV, p = 0.0414, Fig. 1a). Linear discriminant analysis effect size (LEfSe) indicated several *Clostridiales* had lower abundance at T3 compared to baseline (including *Ezakiella, Peptoniphilus*).

### The anal microbiome

**Figure 2:**
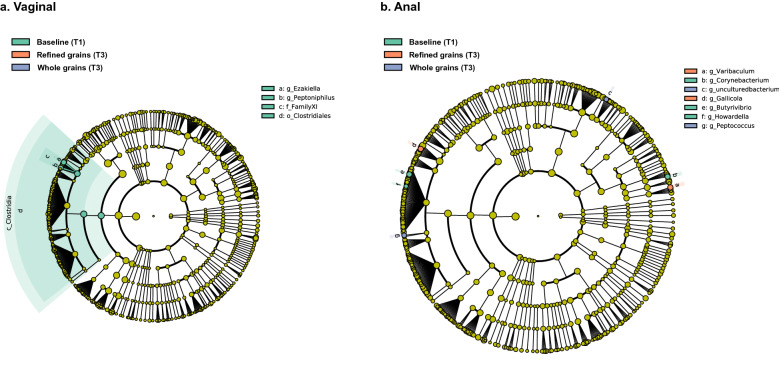
LEfSe cladogram of differing taxa. Significantly enriched taxa colored in purple for refined grains diet, green for whole grains diet. g_ for genus level, f_ for family level, o_ for order level, c_ for class level, and p_ for phylum level. (**a**) Vaginal overrepresented taxa in each group. (**b**) Anal overrepresented taxa in each group.

**Figure 3:**
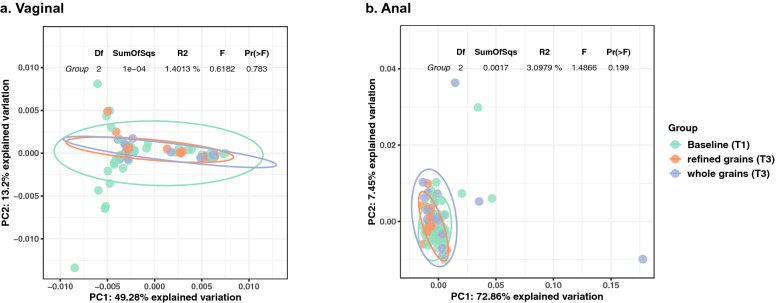
Vaginal and anal microbiome beta diversity. PCoA generated on weighted Unifrac distance. (**a**) Vaginal, (**b**) anal. Table shows the PERMANOVA result effect between baseline, whole grains T3, and refined grains T3. Ellipses represent 95% CI of center of each group under multivariate t-distribution.

The anal microbiome was dominated by *Lactobacillus*, *Prevotella*, *Ezakiella*, *Finegoldia*, and *Bacteroides*, and there were also many low abundance (< 1%) taxon which contributed to about 20% of the mean relative abundance (Supplementary Fig. 2b). LEfSe analysis showed changes in the microbiota of women consuming whole grains diet, with higher abundance of *Peptococcus*, while the refined grains diet group had higher abundance of *Corynebacterium, Butyrivibrio, and Howardella.* For both diet groups, *Varibaculum* and *Gallicola* were lower compared to T1 (Fig. 2b). The anal microbiome showed no significant changes in alpha diversity between the dietary groups and no significant differences in beta diversity were observed by diet (Fig. 3b).

### Clinical results

There was no significant difference between diet groups with respect to age, body mass index, race, and ethnicity at enrollment (Table 1) in the subpopulation from whom vaginal and anal samples were taken. There were differences in the consumption of components of total grains such as increased sweets in the refined grains diet or increased fiber in the whole grains diet. The important observation was that there were differences in the consumption of whole grains as intended where patients on the whole grains diet consumed 78% more whole grains than refined grains diet patients (p = 0.0005), based on FFQ data (Supplementary Table 2). Based on total plasma alkylresorcinol measured on 7 refined grains consumers and 6 for whole grains consumers after at least 3 months on the prescribed diet, the alkylresorcinol data correlated with the findings on FFQ data. The mean plasma levels were 70.4 nmol/L for refined grains and 118.4 nmol/L for whole grains diet, indicating 68% more whole grains consumed in patients on a whole grains diet. The data for total alkylresorcinol and FFQ were consistent with each other, and both showed the expected difference in whole grains consumed based on the specific diets, with the whole grains diet containing 50% more whole grains than the refined grains diet. There were no abnormal glucose tolerance tests, no statistically significant differences between diet groups in maternal weight gain, third trimester blood pressure (Supplementary Table 3), or neonatal outcomes, except for an increased proportion of Cesarean births in patients from the refined grains diet group (Supplementary Table 4). Plausible FFQ data (Supplementary Table 2) was based on a total calories’ range of 1800–5000 kcal/day, and FFQ with total calories outside this range were excluded as implausible given the patient’s observed weight gain. Based on this data, we were able to confirm compliance with both diets in the patients studied.

**Table 1:** Maternal descriptive information at enrollment for microbiome subset.

Descriptive	Refined grains (n = 57)	Whole grains (n = 46)
Average age	28	28
Average BMI	28.1	27
Average gravida	2.5	2.8
Average parity	0.9	1.1
Average # of abortions	0.53	0.46
**Primary language**		
English	35%	33%
Spanish	56%	63%
Both	9%	4%
**Race**		
White	46%	43%
Black	< 1%	< 1%
Asian	< 1%	< 1%
Other	49%	48%
**Ethnicity**		
Hispanic	89%	89%
Non Hispanic	11%	11%

## Discussion

A major concern with performing this study and any dietary interventional study is compliance, particularly over a long period of time. The measures taken in this study to assure compliance were largely successful. In addition, the pregnant patient is highly motivated, and the usual clinical approach includes repeated visits for care including assessing weight gain and dietary counseling. This facilitated reinforcement of compliance to the diets, which was successful as determined by the FFQ and plasma alkylresorcinol measurements in this subset and in the larger population enrolled.

The whole grains diet has been reported to have beneficial effects on healthy non pregnant people, increasing fecal short-chain fatty acids (SCFA)^24^, reducing serum inflammatory markers, such as interleukin 6 and C-reactive protein^25^. This study is the first to study the effect of whole grains diet on vaginal and anal microbiome of pregnant women. The clinical outcomes from the study of the larger cohort of this trial showed no significant difference between the two diets^13^. However, the results of this study, with the current sample size and statistical power show that diet, while not affecting the structure of the intestinal microbiome, marginally affected the gestational dynamics of the vaginal microbiome. In the whole grains diet group, there was a trend of reduced vaginal alpha diversity, during gestation, while in the refined grains group, vaginal diversity remained stable.

A few studies have looked at effect of diet on the vaginal microbial ecosystem^26^. In non-pregnant women, the vaginal microbial diversity and stability are affected by menstrual cycle time, hormonal contraceptive use, exercise level, and diet, with greater diversity in vegetarians^27^. In mice, a high fat diet during pregnancy alters serum steroid and free fatty acid concentrations and lowers vaginal pH^28^. But the dynamic of microbiome during pregnancy is more complicated. The traditional view of the gestational microbiome dynamics is that as gestation progresses, the vaginal alpha diversity is reduced with dominance of *Lactobacillus*^29,30^ mediated by increased estrogen levels that increase vaginal glycogen, a substrate for *Lactobacillus* that converts it into lactic acid thus lowering vaginal pH^30–32^. While a few studies showed the gut^33^ or vaginal^34^ alpha diversity decreases during pregnancy, some studies report that the vaginal/gut microbiomes are stable during pregnancy^35^. In this study, the dynamics of the microbiome during pregnancy seems to be affected by diet. Parity could play a role in gestational dynamics of microbiomes during pregnancy. However, there are limited studies about it. One study in pig showed the parity was linked to several gut bacterial species and affect maternal or offspring gut microbiome^36^. We treated parity as a covariant and in our linear model for alpha diversity or PERMANOVA for beta diversity, and showed no significant effect (supplementary Fig. 3).

Very few studies have assessed diet and the vaginal microbiomes during gestation. Subclinical iron deficiency has been associated with bacterial vaginosis (high diversity), in early pregnancy^37^. Gestational changes in the microbiome are normal and expected to sustain health, possibly related to immune, endocrine, and metabolic states^38^. More studies are needed to determine the mechanisms by which diet modulates the vaginal microbiome.

## Methods

### Ethics

The study protocols and consent forms were reviewed and approved by the New York University Langone Health Institutional Review Board (study # i17-006924). All methods were carried out in accordance with the principles expressed in the Declaration of Helsinki. The trial is registered at ClinicalTrials.gov entitled “Effects of Diet on Pregnancy Outcome and Child Obesity” (NCT03232762, registered on 25/07/2017). All participants provided written informed consent before included.

### Study design

The patients—a total of 303 pregnant mothers- were initially enrolled in the study at 8–23 weeks of gestation and followed at regular intervals for approximately 17 to 32 weeks until delivered at term. Enrollment was not offered after 23 weeks of gestation as it might not be long enough on the prescribed diet to affect outcomes. The newborn infant was evaluated for specific neonatal outcomes by a trained pediatrician. The mother was followed throughout her pregnancy and postpartum by a trained obstetrician. There was randomized sequential assignment of normal healthy pregnant women at the initial clinic visit to refined grains diet (with 75% of carbohydrate calories from refined grains), or to whole grains diet (with 75% of carbohydrate calories from whole grains). Both diets were equal for 2200 total calories and the proportions of carbohydrate, protein and fat as recommended for pregnancy diets by the American College of Obstetricians and Gynecologists^39^. Random number assignment by patient was impractical for counseling purposes, and blinding was not possible. Therefore, randomization of diet assignment by day of initial visit via random number was chosen. If the random number was odd refined grain diet would be selected, if the random number was even whole grains diet was chosen for all patients seen for their initial visit on that day. Exclusion criteria were diagnosis of complications or medical illness as determined by the obstetrician before or during pregnancy.

General dietary instructions were the same for each diet group and every patient had dietary counseling by a registered dietitian or physician at the initial visit and each subsequent clinic session. Spanish and English speakers provided the counseling and instructions in the appropriate language. Each clinic visit was as usual for obstetrical care with the exception of a previously validated Block food frequency questionnaire (NutritionQuest 2018 version) performed on an iPad device^40^. The FFQ food and beverage list includes 127 items, plus additional questions to adjust for fat, protein, carbohydrate, sugar, and whole grain content. The food list for the FFQ was developed from analysis of two waves of National Health and Nutrition Examination Survey (NHANES) dietary recall data. A pregnancy specific diet with ethnically appropriate foods believed to be locally available was selected based on advice of a consultant nutritionist who was a registered dietitian specializing in Hispanic diet counseling. Both the diets followed obstetrical guidelines^39^ and were specific to the assigned diet, refined grains or whole grains diet. Written diet brochures were provided by the research dietitian or the research physician. FFQs were in the appropriate language, administered on an iPad by the research staff fluent in Spanish and English. Food samples were provided at each visit which were appropriate to the patient’s type of grains, but that was to encourage continued participation and the only food supplied. The questionnaires were administered in the third trimester after at least 12 weeks on the diet.

Microbiome samples were collected from 103 mothers who consented for sample collection, a subset of the total patients enrolled, from vaginal and anal sites, using sterile swabs. Vaginal and anal samples were taken when pelvic examinations were clinically indicated. They were at the first visit, before the dietary intervention, clinically indicated for Pap screening and for sexually transmitted disease (T1), and at 34–36 weeks, when group B streptococcus testing was performed routinely (T3). Vaginal samples were taken by inserting a sterile cotton swab no more than 2 inches past the introitus and rotated gently to sample all 4 walls, withdrawn without touching skin and placed into a sterile container. Anal samples were taken by inserting a sterile swab no more than 1.5 in. into the anal canal, gently rotated for 10 s before placed into a sterile container. Samples were kept at – 80 °C until microbiome—16SrRNA survey analysis.

Blood samples for alkylresorcinol to measure plasma levels of whole grain consumption were taken in the third trimester after at least 12 weeks on a diet at the time of routine blood count and glucose screening, only from patients who consented from a subset from the total patients enrolled. Plasma was separated from whole blood and kept at – 80 °C until alkylresorcinol measurements were run. Microbiome samples and blood samples for alkyl resorcinol were labeled with a code assigned to the patients, after they were assigned a diet that de-identified their information, blinding the researchers analyzing those data endpoints.

### Compliance

Brochures with weekly and daily menus with various food choices and estimates of portion size were provided for both diets, in a brochure in Spanish or English, differing only in the whole grains versus refined grains variety of the same food. In week one, each patient had a text message or scripted phone call to report on her diet and the information was recorded as an attempt to assure compliance. Patients were given food samples appropriate for the different diets and again counseled at subsequent monthly visits. Each patient was given the appropriate website for her diet, as well as the recipes, sample menus and the initial diet brochure, all accessible online to assure that they were available even if the print versions were lost. These were interventions for encouraging healthy nutrition and adherence to the assigned diet.

The clinic visits were at the standard monthly frequency of obstetrical care in the regular obstetrical clinics of the hospital. Standard obstetrical measurements were performed at these regular intervals including weight, blood pressure and laboratory tests, and these were recorded and accessed by computer using the Epic system. After 3 months the data was reviewed as a pilot study to include 30 patients in each group. This confirmed the feasibility of the approach.

### Number of subjects

For statistical power calculations, assuming average weight gain in pregnancy of 15 kg for women aged 25–29^41,42^ with normal BMI, we chose to detect a 20% difference in average weight gain (3 kg), as our primary maternal endpoint. We needed to study 96 refined grain diet subjects and 96 whole grains diet subjects to be able to reject the null hypothesis with probability 0.05. Statistical analysis was by non-parametric Mann–Whitney U test. The number of samples from the examined subpopulation was based on previous reports by MacIntyre^32^ and Dominguez-Bello^43^.

### Subjects

All were healthy pregnant women age 18–45 with normal pregnancy without medical disease and uncomplicated as defined by the obstetrician. All were taking no medication other than prenatal vitamins and were more than 3 months from term when enrolled. The patients were without private insurance, attending the same Obstetrical Clinic. Spanish was the primary language for the majority, while the rest spoke English.

### Racial and ethnic origin

There was no restriction on inclusion related to race or ethnicity, but the patients were primarily Spanish speakers. Diets were based on this Spanish speaking ethnicity with specific foods accessible locally as chosen by an appropriate consultant.

### Study endpoints

Maternal Outcomes: Weight gain, Weight at term, Blood pressure, Hypertension/pre-eclampsia, Glucose Challenge Test, Gestational Diabetes Diet compliance by FFQ and alkylresorcinol results.

### Analysis of alkylresorcinols

Alkylresorcinols constitute a series of odd numbered 1,3-dihydroxy-5-alkylbenzene homologues with 17–25 carbon atoms in the side chain. These molecules are localized in the bran of wheat and rye among commonly consumed foods but not in the white flour. They are absorbed in relation to intake and can be measured in plasma. Based on this, alkylresorcinols in plasma and their metabolites in plasma and urine have been used as dietary biomarkers of whole grain wheat and rye intake. In the current study, plasma alkylresorcinols were used as biomarkers. Frozen samples were sent blinded, and measurements performed in the laboratory of Professor Rikard Landberg in Sweden^44,45^. The blood sample was obtained between 28 and 36 weeks of gestation after at least 3 months of the prescribed diet, when standard blood tests were drawn for clinical purposes.

### DNA sequencing and analysis

DNA extraction was performed using the QIAGEN DNeasy PowerSoil HTP 96 Kit according to the manufacturer’s instructions. The V4 region of the 16S rRNA gene was amplified using the 515F/806R primers and prepared for sequencing as described in protocols for Earth Microbiome Project (https://earthmicrobiome.org/protocols-and-standards/16s/). Amplicons were pooled in equimolar ratios and were sequenced at GENEWIZ, LLC. (South Plainfield, NJ, USA) using the Illumina MiSeq sequencing instrument.

### Microbiome data analysis

Raw reads were de-multiplexed and quality filtered using QIIME2 v2019.10^46^ using default parameters. The quality-filtered reads were clustered into amplicon sequence variants (ASVs) with DADA2^47^. Taxonomic assignment of the ASVs were obtained by aligning with SILVA database v132. Phylogenetic tree was generated by FastTree algorithm^48^. Negative control samples were also sequenced and ASVs identified as contamination by decontam (R v1.14.0)^49^ were removed from further analysis (N contamination = 15, N total ASV = 2707). Reads count in ASV table were rarefied to 8000 reads per sample. Faith’s Phylogenetic Diversity^50^, observed ASV, and Shannon Index were calculated as alpha diversity metrics. Unweighted/weighted UniFrac distances^51^ were calculated to obtain pairwise beta-diversity and dimensionality reduction on the distances was performed using Principal Coordinates Analysis (PCoA) method (R v4.0.3, ape v5.4-1)^52^. All alpha and beta diversity metrics are calculated on ASV level abundance. Permutational multivariate analysis of variance (PERMANOVA) were used test the significance of different groups with 999 permutations (R v4.0.3, vegan v2.5-7)^53^. Linear discriminant analysis effect size (LEfSe) method^54^ was used to search for differentiated bacterial taxa by groups, i.e. diet.

## Supplementary Information

Supplementary Information 1.

Supplementary Figure 1.

Supplementary Figure 2.

Supplementary Figure 3.

## Data Availability

The 16SrRNA sequences from vaginal and anal samples have been deposited in European Nucleotide Archive (ENA) under project number PRJEB44513.

## Abbreviations

BMI: Basal metabolic index
GCT: Glucose challenge test
GTT: Glucose tolerance test
FFQ: Food frequency questionnaire
