# Supplementary material for: A randomized controlled trial of the effects of whole grains versus refined grains diets on the microbiome in pregnancy
Source: Sci Rep. 2022 May 7;12:7509. doi: 10.1038/s41598-022-11571-4 (PMC9079079; doi:10.1038/s41598-022-11571-4)
Supplement: Supplementary file 1 — Supplementary Information 1. [file 41598_2022_11571_MOESM1_ESM.docx]

**A randomized controlled trial of the effects of whole grains versus refined grains diets on the microbiome in pregnancy**

Haipeng Sun^1^, Pamella Yamada^2^, Alexandra Paetow^2^, Michael Chan^2^, Alan Arslan^2^, Rikard Landberg^3^, Maria Gloria Dominguez-Bello^1,4,5^ and Bruce K. Young^2*^

^1^ Department of Biochemistry and Microbiology, Rutgers University

^2^ New York University Langone Health, Department of Obstetrics and Gynecology

^3^ Chalmers University of Technology, Department of Biological Engineering, Division of Food and Nutrition Science (RL)

^4^ Department of Anthropology, Rutgers University

^5^ Institute of Food, Nutrition, and Health, Rutgers University

^*^Corresponding Author: Bruce K Young, MD, NYU Langone Health Department of Obstetrics and Gynecology, 462 First Avenue, Building D, Room 572, New York, NY 10016; 212-263-6359; [Bruce.Young@nyulangone.org](mailto:Bruce.Young@nyulangone.org)

**Supplementary Figure Legends**

**Supplementary Figure 1. Sampling times of all subjects provided microbiome samples.** Gray dot indicate before intervention, baseline (T1) (GA week 5 -26) and dark green (whole grains group) and dark purple (refined grains group) for the second time point, T3 (GA week 32- 40). Each row along y-axis represents an individual subject. The same subject was linked with line if provide samples for both time points.

**Supplementary Figure 2.** **Vaginal and anal mean Relative abundance of dominant genera.** Genera with relative abundance less than 1% are grouped in other. **a** vaginal microbiome, **b** anal microbiome.

**Supplementary Figure 3. Effect of Gravidity on vaginal and anal microbiome.** Alpha diversity by gravidity, Faith PD, Observed ASVs, and Shannon index was presented respectively. **a** vaginal, **b** anal. Table shows effect of alpha diversity by diet, time point, and gravidity in a linear model. PCoA generated on weighted Unifrac distance. **c** vaginal, **d** anal. Table shows the PERMANOVA result of a mixed model of diet, time point, and gravidity.

**Supplementary Table 1 Number of subjects and samples for microbiome analyses.**

|  | **T1 only** | **T3 only** | **Sequential** |
| --- | --- | --- | --- |
| **Total number of subjects** | 58 | 39 | 6 |
| **Refined Grains** | 35 | 19 | 3 |
| **Whole Grains** | 23 | 20 | 3 |
| **Total number of anal samples** | 58 | 39 | 12 |
| **Refined Grains** | 35 | 19 | 6 (3 T1 and 3 T3) |
| **Whole Grains** | 23 | 20 | 6 (3 T1 and 3 T3) |
| **Total number of vaginal samples** | 58 | 38 | 12 |
| **Refined Grains** | 35 | 19 | 6 (3 T1 and 3 T3) |
| **Whole Grains** | 23 | 19 | 6 (3 T1 and 3 T3) |

**Supplementary Table 2** **Dietary data from** **food frequency questionnaire.**

|  | **Food Frequency Questionnaire** | **Refined Grains** | **Standard Deviation** | **Whole Grains** | **Standard Deviation** | **Statistical Significance*** |
| --- | --- | --- | --- | --- | --- | --- |
|  |  |  |  |  |  |  |
|  | **Calories** | 1844.6  (n=36) | 717.9 | 2,107.9  (n=32) | 983.7 | p=0.25 |
|  |  |  |  |  |  |  |
|  | **Fat (grams)** | 75.6  (n=36) | 30.5 | 81.3  (n=32) | 38.2 | p=0.67 |
|  | **Protein (grams)** | 69.2  (n=36) | 29.3 | 80.4  (n=32) | 41.4 | p=0.23 |
|  | **Carbohydrates**  **(grams)** | 232.3  (n=36) | 95.3 | 276.0  (n=32) | 137.1 | p=0.17 |
|  | **Fiber (grams)** | 22.9  (n=36) | 10.9 | 27.5  (n=32) | 14.5 | p=0.19 |
|  |  |  |  |  |  |  |
|  | **Sweets (% of kcal)** | 10.4  (n=36) | 9.4 | 39.3  (n=32) | 15.8 | p=0.31 |
|  | **Bread, pasta, rice**  **(grams)** | 159  (n=36) | 73.0 | 218.7  (n=32) | 107.9 | p=0.02 |
|  | **Whole grains**  **(grams)** | 25.6  (n=36) | 28.4 | 45.4  (n=32) | 28.4 | p=0.0005 |

^*^p-value determined by non-parametric Mann-Whitney U test

**Supplementary Table 3 Gestational weight gain and morphometric measurements for microbiome subset**

| Maternal Measurements | Refined Grains | Standard Deviation | Whole Grains | Standard Deviation | Statistical Significance* |
| --- | --- | --- | --- | --- | --- |
|  |  |  |  |  |  |
| **Weight Gain (kg)** | **10.5**  **(n=52)** | **4.6** | **10.2**  **(n=38)** | **4.7** | **p=0.68** |
| **Weight at term (kg)** | **75.4**  **(n=52)** | **14.3** | **74.8**  **(n=38)** | **14.3** | **p=0.72** |
|  |  |  |  |  |  |
| **1^st^ Trimester Systolic Blood Pressure**  **(mm Hg)** | **109.6**  **(n=57)** | **8.3** | **107.5**  **(n=46)** | **10.2** | **p=0.29** |
| **1^st^ Trimester Diastolic Blood Pressure**  **(mm Hg)** | **71.1**  **(n=57)** | **6.4** | **68.5**  **(n=46)** | **6.5** | **p=0.12** |
| **3^rd^ Trimester Systolic Blood Pressure**  **(mm Hg)** | **112.8**  **(n=52)** | **11.0** | **112.1**  **(n=36)** | **11.2** | **p=0.44** |
| **3^rd^ Trimester Diastolic Blood Pressure**  **(mm Hg)** | **71.0**  **(n=52)** | **8.5** | **69.6**  **(n=36)** | **7.4** | **p=0.21** |

^*^p-value determined by non-parametric Mann-Whitney U tes

**Supplementary Table 4 Neonatal outcomes for each diet group.**

| **Neonatal Outcomes** | **Refined grains** | **Standard Deviation** | **Whole grains** | **Standard Deviation** | **Statistical Significance** |
| --- | --- | --- | --- | --- | --- |
|  |  |  |  |  |  |
| **Weeks at Delivery** | 38.9  (n=52) | 1.0 | 39.3  (n=39) | 1.3 | ^*^p=0.03 |
| **Neonatal Weight (grams)** | 3,362.1  (n=51) | 500.8 | 3,461.5  (n=36) | 311.0 | ^*^p=0.22 |
| **Crown-heel length (cm)** | 50.3  (n=24) | 2.4 | 50.9  (n=12) | 1.5 | ^*^p=0.64 |
| **Apgar score at 1 min** | 8.7  (n=32) | 0.5 | 8.4  (n=20) | 1.7 | ^*^p=0.52 |
| **Apgar score at 5 min** | 9.0  (n=32) | 0.2 | 8.9  (n=20) | 0.7 | ^*^p=0.73 |
|  |  |  |  |  |  |
| **Delivery Mode** |  |  |  |  |  |
| **C-section (%)** | 31.6  (n=18) |  | 19.6  (n=9) |  | ^+^p=0.34 |
| **Normal Spontaneous Delivery (%)** | 57.9  (n=33) |  | 65.2  (n=30) |  |  |
| **Vacuum (%)** | 1.7  (n=1) |  | 0.0  (n=0) |  |  |
| **Not stated (%)** | 8.8  (n=5) |  | 15.2  (n=7) |  |  |

^*^p-value determined by non-parametric Mann-Whitney U test

^+^p-value determined by Chi-Square test
