# Supplementary figures and images for: A randomized controlled trial of the effects of whole grains versus refined grains diets on the microbiome in pregnancy
Source: Sci Rep. 2022 May 7;12:7509. doi: 10.1038/s41598-022-11571-4 (PMC9079079; doi:10.1038/s41598-022-11571-4)

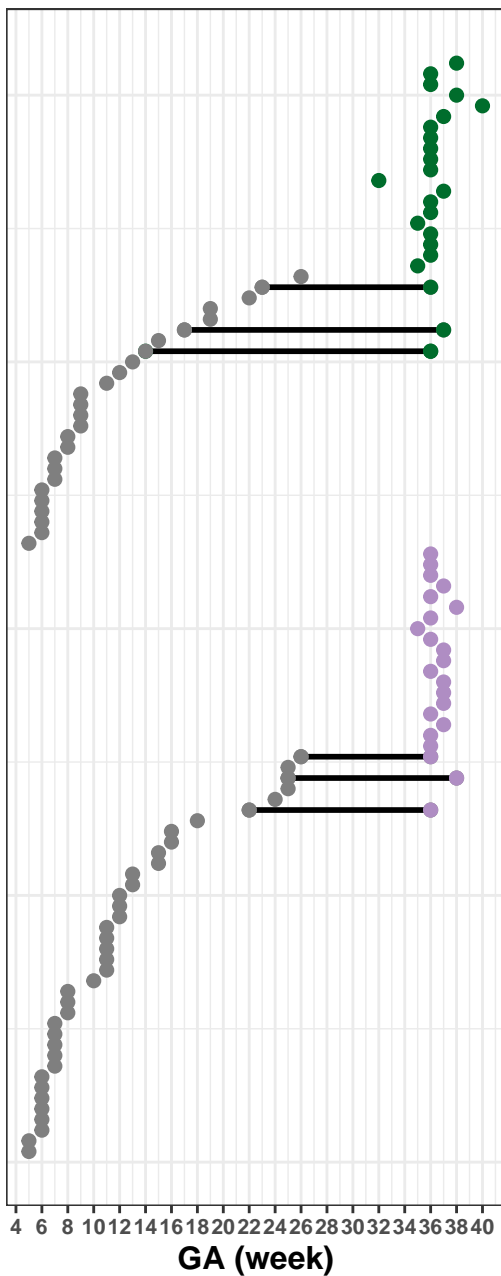

Supplement: Supplementary file 2 — Supplementary Figure 1. [file 41598_2022_11571_MOESM2_ESM.pdf]

a. Vaginal

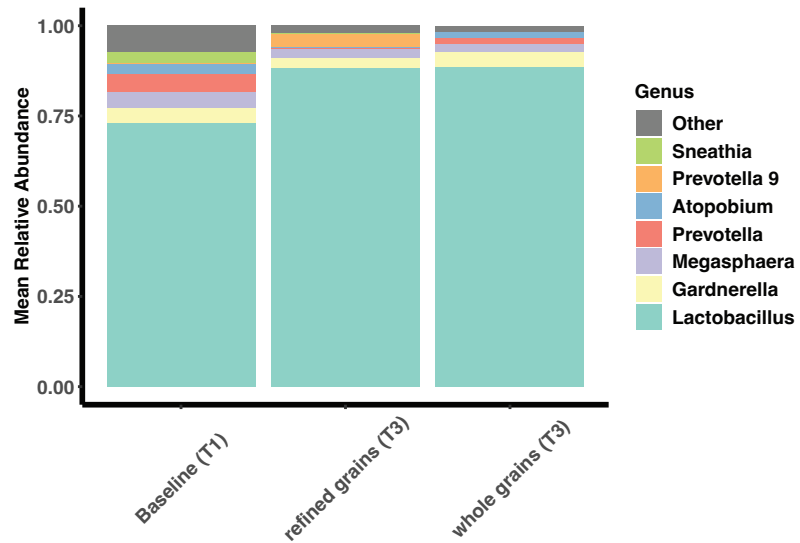

b. Anal

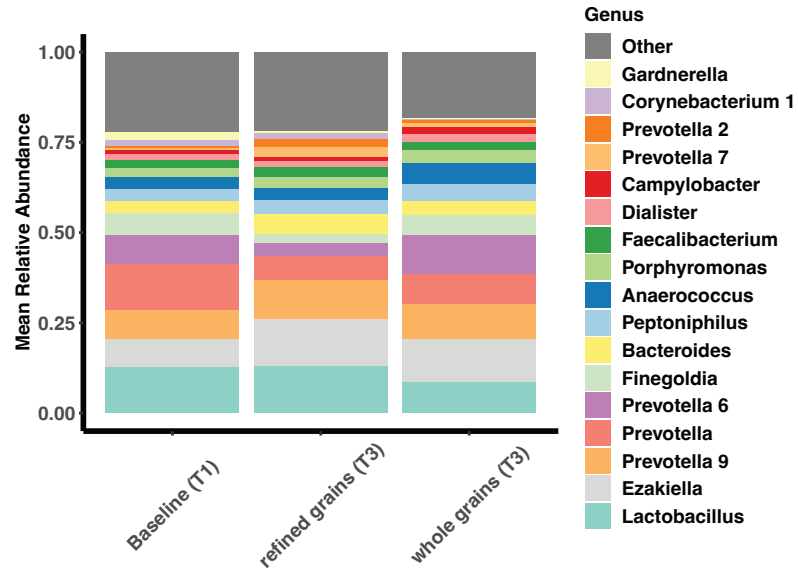

Supplement: Supplementary file 3 — Supplementary Figure 2. [file 41598_2022_11571_MOESM3_ESM.pdf]
