# Supplementary material for: A randomized controlled trial of the effects of whole grains versus refined grains diets on the microbiome in pregnancy
Source: Sci Rep. 2022 May 7;12:7509. doi: 10.1038/s41598-022-11571-4 (PMC9079079; doi:10.1038/s41598-022-11571-4)
Supplement: Supplementary file 4 — Supplementary Figure 3. [file 41598_2022_11571_MOESM4_ESM.pdf]

a. Vaginal alpha diversity

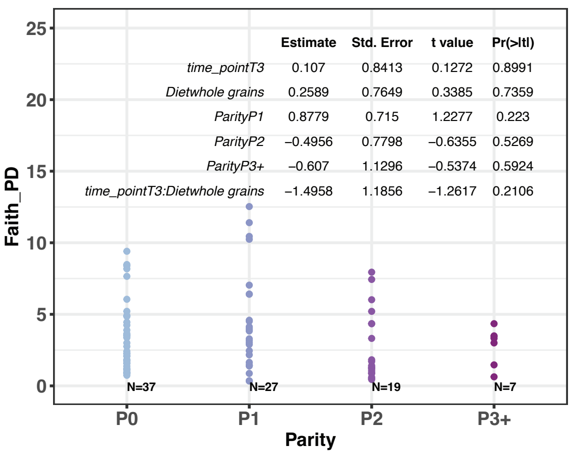

	Estimate	Std. Error	t value	Pr(>|t|)
time_pointT3	0.107	0.8413	0.1272	0.8991
Dietwhole grains	0.2589	0.7649	0.3385	0.7359
ParityP1	0.8779	0.715	1.2277	0.223
ParityP2	−0.4956	0.7798	−0.6355	0.5269
ParityP3+	−0.607	1.1296	−0.5374	0.5924
time_pointT3:Dietwhole grains	−1.4958	1.1856	−1.2617	0.2106

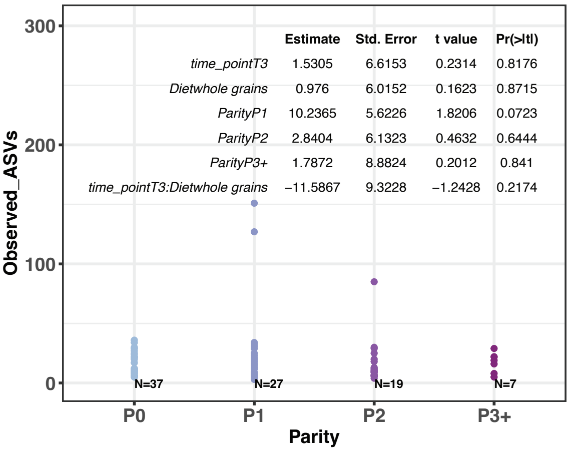

	Estimate	Std. Error	t value	Pr(>|t|)
time_pointT3	1.5305	6.6153	0.2314	0.8176
Dietwhole grains	0.976	6.0152	0.1623	0.8715
ParityP1	10.2365	5.6226	1.8206	0.0723
ParityP2	2.8404	6.1323	0.4632	0.6444
ParityP3+	1.7872	8.8824	0.2012	0.841
time_pointT3:Dietwhole grains	−11.5867	9.3228	−1.2428	0.2174

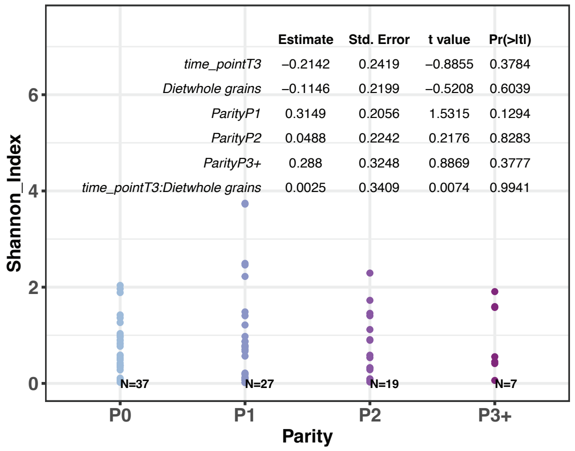

	Estimate	Std. Error	t value	Pr(>|t|)
time_pointT3	−0.2142	0.2419	−0.8855	0.3784
Dietwhole grains	−0.1146	0.2199	−0.5208	0.6039
ParityP1	0.3149	0.2056	1.5315	0.1294
ParityP2	0.0488	0.2242	0.2176	0.8283
ParityP3+	0.288	0.3248	0.8869	0.3777
time_pointT3:Dietwhole grains	0.0025	0.3409	0.0074	0.9941

b. Anal alpha diversity

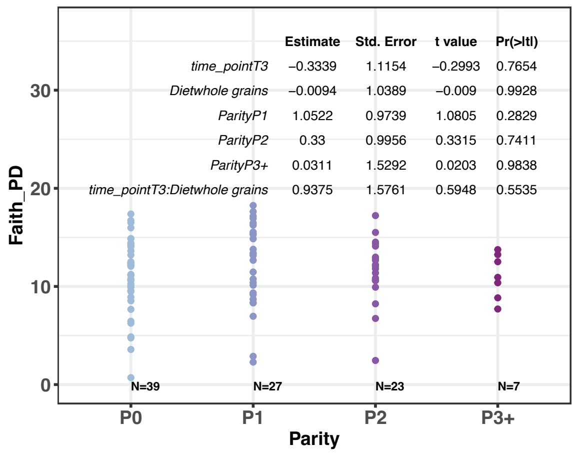

	Estimate	Std. Error	t value	Pr(>|t|)
time_pointT3	−0.3339	1.1154	−0.2993	0.7654
Dietwhole grains	−0.0094	1.0389	−0.009	0.9928
ParityP1	1.0522	0.9739	1.0805	0.2829
ParityP2	0.33	0.9956	0.3315	0.7411
ParityP3+	0.0311	1.5292	0.0203	0.9838
time_pointT3:Dietwhole grains	0.9375	1.5761	0.5948	0.5535

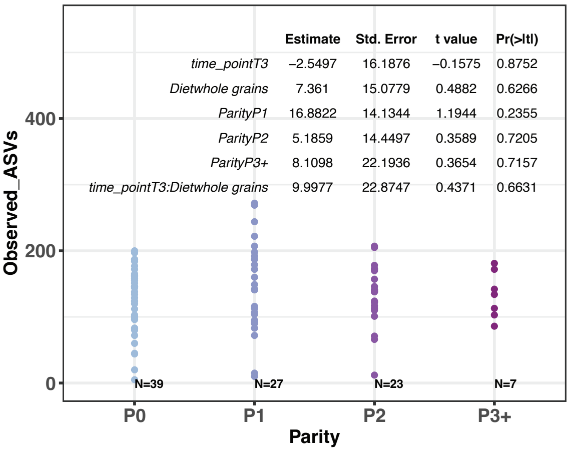

	Estimate	Std. Error	t value	Pr(>|t|)
time_pointT3	−2.5497	16.1876	−0.1575	0.8752
Dietwhole grains	7.361	15.0779	0.4882	0.6266
ParityP1	16.8822	14.1344	1.1944	0.2355
ParityP2	5.1859	14.4497	0.3589	0.7205
ParityP3+	8.1098	22.1936	0.3654	0.7157
time_pointT3:Dietwhole grains	9.9977	22.8747	0.4371	0.6631

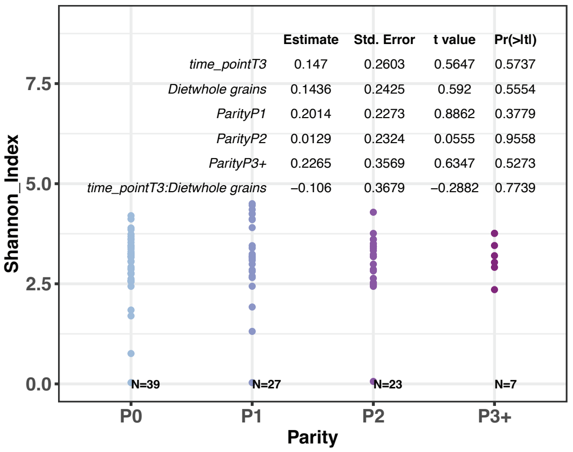

	Estimate	Std. Error	t value	Pr(>|t|)
time_pointT3	0.147	0.2603	0.5647	0.5737
Dietwhole grains	0.1436	0.2425	0.592	0.5554
ParityP1	0.2014	0.2273	0.8862	0.3779
ParityP2	0.0129	0.2324	0.0555	0.9558
ParityP3+	0.2265	0.3569	0.6347	0.5273
time_pointT3:Dietwhole grains	−0.106	0.3679	−0.2882	0.7739

c. Vaginal beta diversity

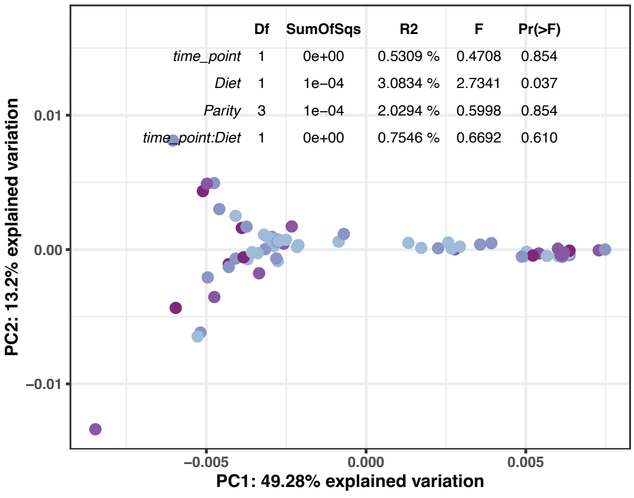

	Df	SumOfSqs	R2	F	Pr(>F)
time_point	1	0e+00	0.5309 %	0.4708	0.854
Diet	1	1e−04	3.0834 %	2.7341	0.037
Parity	3	1e−04	2.0294 %	0.5998	0.854
time_point:Diet	1	0e+00	0.7546 %	0.6692	0.610

d. Anal beta diversity

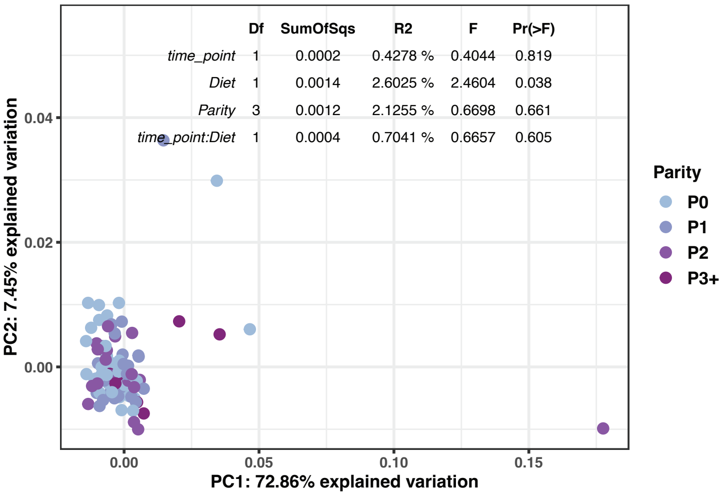

	Df	SumOfSqs	R2	F	Pr(>F)
time_point	1	0.0002	0.4278 %	0.4044	0.819
Diet	1	0.0014	2.6025 %	2.4604	0.038
Parity	3	0.0012	2.1255 %	0.6698	0.661
time_point:Diet	1	0.0004	0.7041 %	0.6657	0.605
